# Reply to comment on: Patellar instability in Indian population: relevance of tibial tuberosity and trochlear groove distance: SICOT J 2019, **5**, 24, https://doi.org/10.1051/sicotj/2019018

**DOI:** 10.1051/sicotj/2019014

**Published:** 2019-07-24

**Authors:** Sourabh Kulkarni, Amith P. Shetty, Karan K. Alva, Saurabh Talekar, Vijay D. Shetty

**Affiliations:** Hiranandani Orthopaedic Medical Education (HOME), Dr L. H. Hiranandani Hospital Hillside Avenue, Hiranandani Gardens Powai, Mumbai 400076 India

To,

The editor

First of all please convey many thanks to author of “Letter to editor” for his interest in our paper [1]. As per the conclusion we have two questions to be answered:

1. Whether the measured parameter should be called as “Patellar tendon–Trochlear Groove distance (PT–TG Distance)” or “Tibial tuberosity–Trochlear Groove Distance (TT–TG Distance)”;
2. Methodology used in the study needs to be verified.

It is a very well known fact that any parameter can have more than one method to measure it. Results obtained by one method may differ from another to an extent. But that does not undermine the importance of the actual parameter or does not necessitate to rename it every time when it is measured by a different method. If with each method we change the terminology of that parameter, then confusion is inevitable and importance of the parameter in discussion may fade away.

According to literature, while measuring TT–TG Distance multiple methods are used to define tibial tuberosity and trochlear groove.

To determine tibial tuberosity, the following landmarks are used in various studies:

1. Anterior most point of tibial tuberosity (bony landmark) [2];
2. Center of tibial tubercle (bony landmark);
3. Center of distal most part of patellar tendon attached over tibial tuberosity (cartilaginous/soft tissue landmark) [3, 4].

On the trochlear side,

1. The deepest portion of the bony trochlear groove (bony landmark) [2];
2. The deepest portion of the cartilaginous trochlear groove (cartilaginous/ soft tissue landmark).

Also there are multiple studies with contradicting results regarding accuracy of bony vs cartilaginous landmarks and also regarding use of CT scan vs. MR imaging as imaging modalities [5, 6].

Even though the landmarks varied in multiple studies, many of them have maintained a discipline to refer measured parameter as TT–TG Distance [3, 4].

We preferred to refer this measurement as TTTG distance considering following points:

1. It is a well-known fact that tibial tuberosity has its significance in discussion because it has patellar tendon attached over it. In other words, tibial tubercle without patellar tendon attachment has no clinical significance and should not be considered for measurement. So we took part of tibial tuberosity for measurement which has patellar tendon attached over it and referred it as “tibial tuberosity (TT)”. We did not refer it as “patellar tendon (PT)” because we are measuring the distance from clinically significant tibial tuberosity and not from the patellar tendon.
2. Multiple studies have used the term “TTTG distance” for the same measurement methodology that we used. On the contrary, very few recent articles have come up with newly coined terminology as “PT–TG Distance” for this method. We decided to go by traditional and widely accepted terminology TT–TG distance and not PT–TG distance.
3. In literature we found that two different measurements are being referred as PTTG distance:
  - patellar tip to trochlear groove distance (PTTG distance) [7];
  - Patellar tendon to trochlear groove distance (PTTG distance).

So we wanted to avoid confusion.

But we will have no objection to refer this measurement as PTTG distance instead of TTTG distance if

- The method used by us to measure the said parameter is unanimously accepted by orthopedic community as PTTG distance for future reference and research;
- The confusion about PT–TG distance terminology is resolved.

Now coming to the second question to be answered, there are studies including ours in which the good inter-observer reliability for the measurement has been found. This should resolve the doubt in the methodology.

Hope all the queries are answered to satisfaction.
